# Association between cumulative exposure to adverse childhood experiences and childhood obesity

**DOI:** 10.1371/journal.pone.0239940

**Published:** 2020-09-29

**Authors:** Pooja Purswani, Sarah M. Marsicek, Ernest K. Amankwah

**Affiliations:** 1 Department of Medicine, Johns Hopkins All Children's Hospital, St. Petersburg, Florida, United States of America; 2 Department of Oncology, Johns Hopkins University School of Medicine, Baltimore, Maryland, United States of America; 3 Cancer and Blood Disorders Institute, Johns Hopkins All Children’s Hospital, St. Petersburg, Florida, United States of America; Hong Kong Polytechnic University, HONG KONG

## Abstract

**Background:**

Exposure to adverse childhood experiences (ACEs) is associated with many childhood diseases and poor health outcomes in adulthood. However, the association with childhood obesity is inconsistent. We investigated the association between reported cumulative ACE score and body mass index (BMI) in a large sample of patients at a single institution.

**Methods:**

This cross-sectional study included children aged 2–20 years that were screened in a general pediatrics clinic for ACEs utilizing the Center for Youth Wellness ACEs questionnaire between July 2017 and July 2018. Overall ACE score was categorized as ‘no exposure’ (score = 0), ‘low exposure’ (score = 1), and ‘high exposure’ (score≥ 2). BMI was categorized as overweight/obese (BMI percentile ≥ 85) or non-obese (BMI percentile < 85). The association between ACEs score and obesity was determined using univariate and multivariable logistic regression.

**Results:**

Of the 948 patients included in the study, 30% (n = 314) were overweight/obese and 53% (n = 504) had no ACE exposure, 19% (n = 179) had low ACE exposure, and 28% (n = 265) had high ACE exposure. High ACE exposure was associated with increased odds of obesity (OR = 1.47, 95%CI = 1.07–2.03, p = 0.026). However, after adjusting for age, race/ethnicity, insurance type, and birth weight, the association attenuated and was null (OR = 1.01, 95%CI = 0.70–1.46, p = 0.97).

**Conclusion:**

The study findings may suggest an association between ACE and childhood obesity. However, the association attenuated after adjusting for age, race/ethnicity, insurance type, and birth weight. Larger prospective studies are warranted to better understand the association.

## Introduction

Adverse Childhood Experiences (ACEs), a term coined by Felitti and colleagues in 1998, refers to psychological, physical, or sexual abuse and household dysfunction experienced by youth under the age of eighteen [1]. In their landmark study, “The Adverse Childhood Experiences Study”, they described a dose-response relationship between exposure to traumatic or emotionally distressing experiences during childhood and the development of chronic health conditions in adulthood, such as heart disease, chronic respiratory disease, cancer, and diabetes [1]. Since then, the presence of multiple childhood exposures have also been associated with adult health risk behaviors, such as alcohol and substance use disorders, as well as mental disorders and obesity in adults and well-being throughout the life span [2, 3].

The association between ACE and obesity is plausible. Parental incarceration and child maltreatment are associated with poor dietary and sleep practices including sweets, soda and salty snack consumption and short sleep duration [4, 5]. These poor dietary and sleep practices are associated with obesity [6]. Similarly, ACE affects telomere length [7, 8], which is associated with obesity [9, 10]. In addition, food insecurity is associated with obesity in adults [11] and a recent study showed that a high cumulative adverse childhood experiences is associated with food insecurity [12].

Emerging evidence in the last decade also suggests that childhood adversity negatively affects pediatric health, including obesity. Children with separated or divorced parents, mothers exposed to intimate partner violence or children who lived in a dysfunctional household were more likely to be overweight [13]. In addition, adolescent females exposed to maltreatment or had been sexually abused were more likely to be obese [14, 15].

These previous studies are informative, however the majority of them examined individual aspects or a limited number of ACEs. It is known that the risk of negative outcomes associated with adverse experiences is more pronounced in individuals who experience multiple ACEs [3, 16, 17]. Presently, there is a paucity of knowledge on the cumulative effects of ACEs on childhood obesity. Understanding this association, could ultimately guide future interventions that might have a positive, life-long influence on a child’s overall health and well-being.

In July 2017, as a result of the increasing awareness of the effects of ACEs on our pediatric population, the Johns Hopkins All Children’s Hospital General Pediatric and Adolescent Medicine (GPAM) clinic initiated screening of all children for ACEs at annual well child visits utilizing a questionnaire published by the Center for Youth Wellness [18]. In this analysis, we examined the association between cumulative ACE scores and body mass index (BMI) using data on 1,200 children screened between July 2017 and July 2018.

## Methods

**Fig 1:**
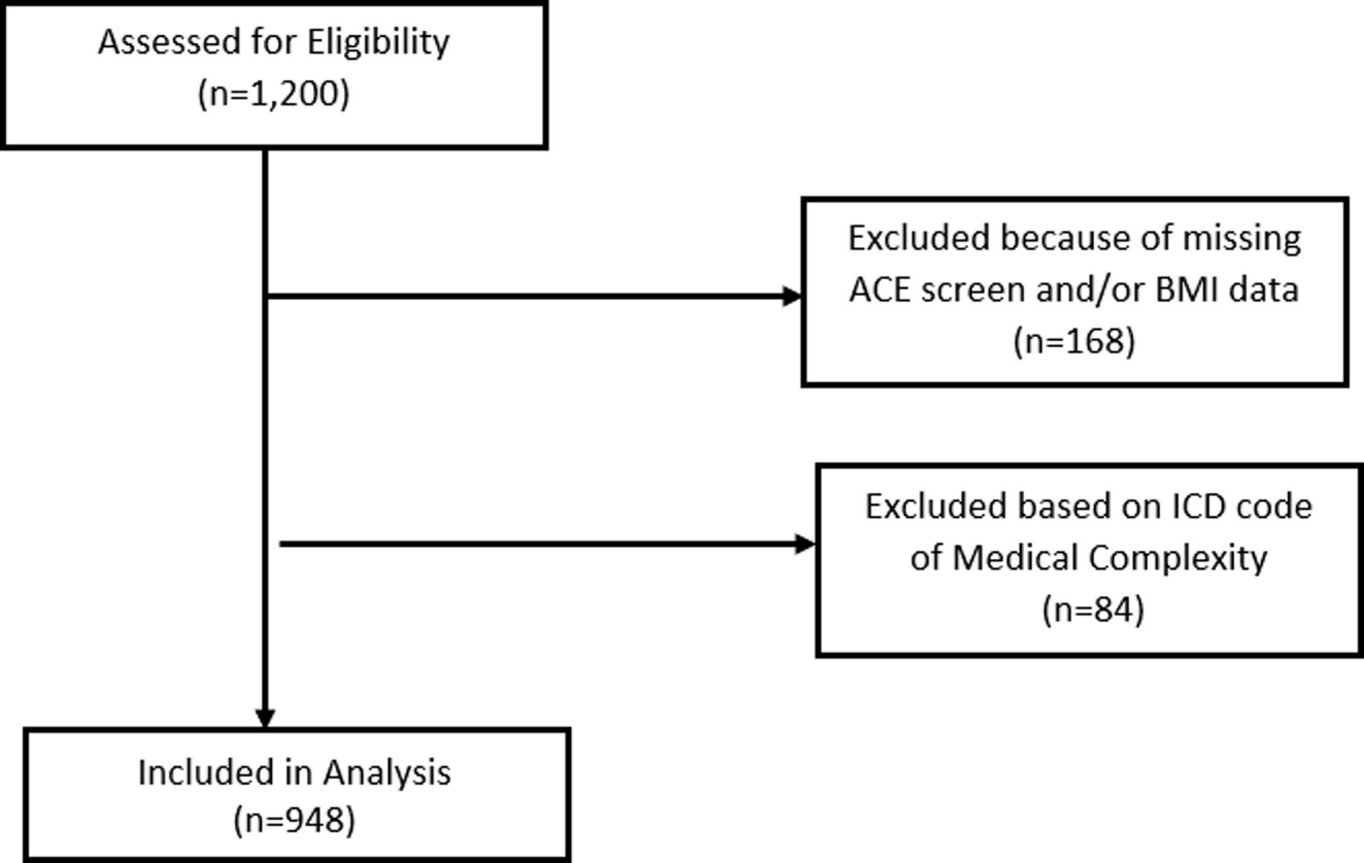
Participant flow diagram. Diagram visually representing the number of participants excluded due to history of medical complexity and incomplete ACE screen and/or BMI data.

This is a cross-sectional study that utilized existing data from the electronic health record-derived Data Warehouse (EHR-DW) at Johns Hopkins All Children’s Hospital between July 2017 and July 2018. The study included children and adolescents, aged 2–20 years, presenting for well child visits at the Johns Hopkins All Children’s Hospital General Pediatric and Adolescent Medicine (GPAM) who were screened for ACEs (n = 1,200) (Fig 1). Patients were excluded if they did not have a completed ACEs screener or BMI obtained during the same visit (n = 168). Additionally, patients with a medically complex diagnosis code (ICD Z78.9) were excluded (n = 84), because this diagnosis code is usually used to identify patients who typically have chronic and/or severe conditions, functional limitations, and may be technology dependent for activities of daily living [19]. The final analytic dataset had 948 participants.

The primary outcome variable was obesity status based on BMI. BMI obtained from the data warehouse were used to determine age-adjusted BMI percentiles using the 2000 Centers for Disease Control and Prevention BMI for age growth charts for children between 2 and 20 years of age (www.cdc.gov/growthcharts). Due to small numbers in some groups, participants were classified as obese by combining overweight/obese (≥85^th^ percentile) and as non-obese (<85^th^ percentile). ACEs score was the main exposure variable. A child’s ACE score was obtained in the clinic using an existing questionnaire developed and published by Center for Youth Wellness [18]. This questionnaire includes 10 ACEs described in the Adverse Childhood Experiences Study as well as other additional early life stressors identified by childhood adversity experts, including living in a foster care, being bullied, death of a caregiver, deportation or migration, discrimination, experiencing a life-threatening illness or invasive medical procedure, and, exposure to community or school violence [1, 20].

As a tool designed to calculate the cumulative exposure to adverse experiences, parents are asked to count the number of ACEs their child has been exposed to and to write the total number on the form. The total ACE score was categorized as ‘no ACE exposure’ (ACE score = 0), ‘low ACE exposure’ (ACE = 1), and ‘high ACE exposure’ (ACE≥ 2) as previously reported [21]. We also obtained, from the data warehouse, other demographic data, including age, gender, race/ethnicity, insurance status, birthweight and history of prematurity, which may increase a child’s risk for obesity. The study was approved by the Johns Hopkins All Children's Hospital Institutional Review Board, with a waiver of informed consent.

### Statistical analysis

Demographic characteristics of the study participants were summarized using counts and percentages for categorical variables and mean (standard deviation) or median (range) for continuous variables. The association between ACEs score, as continuous or categorical variable, and obesity was determined using univariate and multivariable logistic regression. Variables that were screened as potential confounders included age, gender, race/ethnicity, insurance status, birthweight and history of prematurity. A variable was included in the multivariable model if it had a p-value<0.1 in a univariate model. All statistical analyses were performed using SAS v 9.4 and a p-value <0.05 was considered statistically significant.

## Results

**Fig 2:**
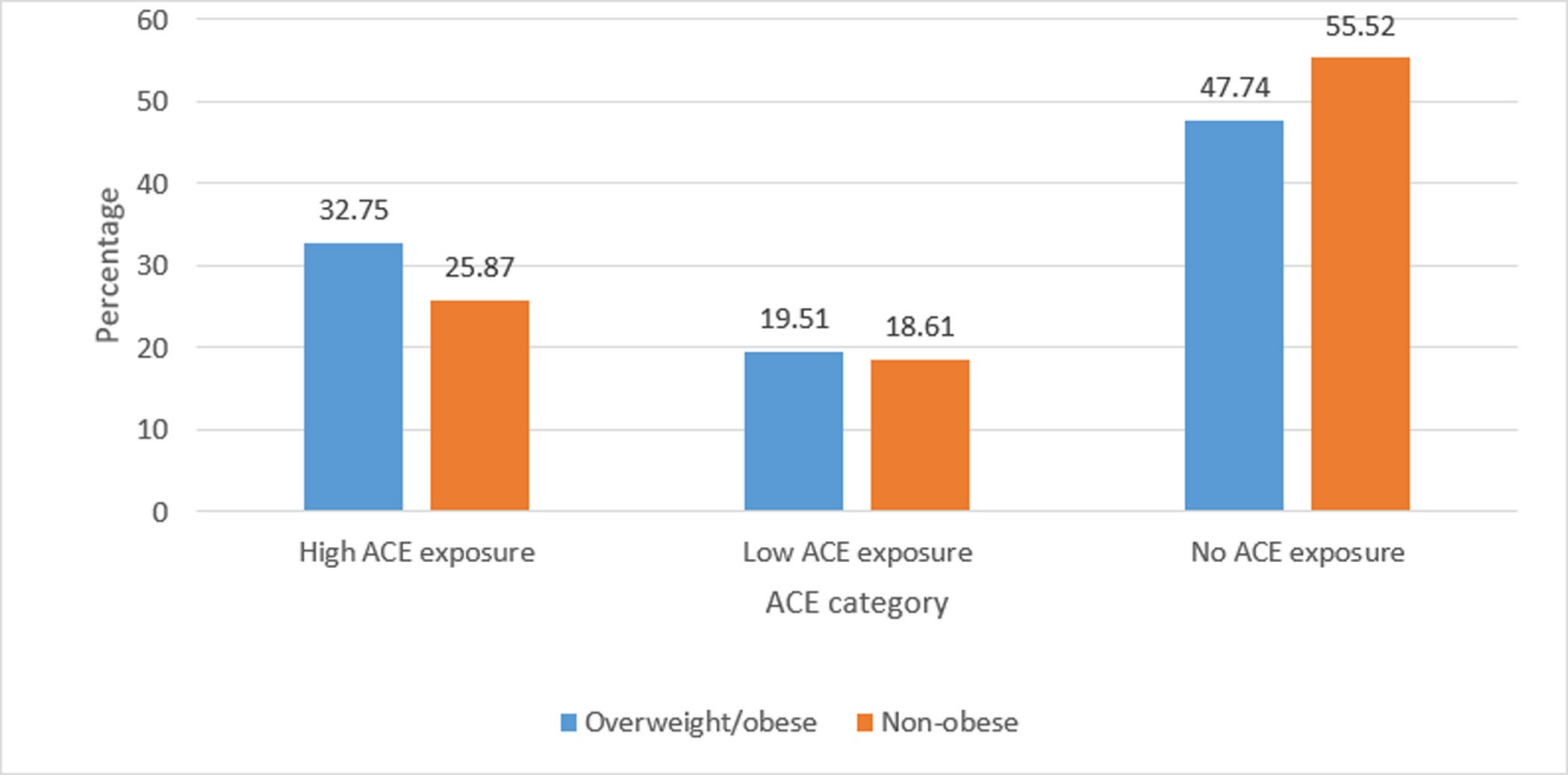
Prevalence of ACE by obesity status: Figure demonstrates percentage of individuals who were overweight and obese by category of ACE exposure.

Characteristics of the study population are presented in Table 1. Of the 948 patients included in the study, 48% (n = 499) were females. The median (range) age was 7 (2–19) years. The proportion of Non-Hispanic Blacks (37%, n = 353) or Non-Hispanic Whites (37%, n = 351) was higher than Hispanics (12%, n = 112). The majority of the study participants did not have a history of prematurity (93%) or low birthweight (94%). Approximately 30% (n = 314) of the participants were overweight/obese and 52% (n = 540) had no ACE exposure, 19% (n = 201) had low ACE exposure, and 291 (28%) had high ACE exposure. The prevalence of high ACE exposure among overweight/obese participants (33%) was higher than that of non-obese participants (26%) (Fig 2).

**Table 1:** Characteristics of study participants by obesity status.

Covariate		Level	Overweight/Obese N = 287	Non-Obese N = 661
Age (Years)	N		287	661
	Mean (SD)		9.08 (4.78)	7.2 (4.59)
	Median (IQR)		9 (5–13)	6 (3–10)
Age category (years)	N (Col %)	0–5	88 (30.66)	323 (48.87)
	N (Col %)	6–12	120 (41.81)	220 (33.28)
	N (Col %)	13+	79 (27.53)	118 (17.85)
Gender	N (Col %)	Female	141 (49.13)	327 (49.47)
	N (Col %)	Male	146 (50.87)	334 (50.53)
Race/ethnicity	N (Col %)	Hispanic	41 (16.08)	61 (10)
	N (Col %)	Non-Hispanic Black	101 (39.61)	225 (36.89)
	N (Col %)	Non-Hispanic White	78 (30.59)	239 (39.18)
	N (Col %)	Other	35 (13.73)	85 (13.93)
Insurance type	N (Col %)	Other	6 (2.09)	12 (1.82)
	N (Col %)	Private	76 (26.48)	227 (34.34)
	N (Col %)	Public	205 (71.43)	422 (63.84)
premature	N (Col %)	No	275 (95.82)	620 (93.8)
	N (Col %)	Yes	12 (4.18)	41 (6.2)
Low Birth Weight	N (Col %)	No	279 (97.21)	624 (94.4)
	N (Col %)	Yes	8 (2.79)	37 (5.6)
ACE	Median (range)		1 (0–12)	0 (0–15)
ACE category	N (Col %)	High ACE exposure	94 (32.75)	171 (25.87)
	N (Col %)	Low ACE exposure	56 (19.51)	123 (18.61)
	N (Col %)	No ACE exposure	137 (47.74)	367 (55.52)

In univariate analysis, high ACE exposure was associated with increased odds of obesity (OR = 1.47, 95%CI = 1.07–2.03, p = 0.017) (Table 2). Low ACE exposure was also associated with a slight increase in the odds of obesity, but the association was not statistically significant (OR = 1.22, 95%CI = 0.84–1.77, p = 0.30). Age, race, insurance type and low birth weight met the criterion to be included in the multivariable model. However, after controlling for these potential confounding variables both associations of ACE (High exposure: OR = 1.01, 95%CI = 0.70–1.46, p = 0.97; Low exposure: OR = 1.03, 95%CI = 0.68–1.55, p = 0.89) were null (Table 3).

**Table 2:** Univariate association between obesity and ACE.

Covariate	Level	N	Odds Ratio	OR P-value
			(95% CI)	
ACE continuous		948	1.07 (1.01–1.15)	**0.029**
ACE category	High ACE exposure	265	1.47 (1.07–2.03)	**0.017**
	Low ACE exposure	179	1.22 (0.84–1.77)	0.3
	No ACE exposure	504	-	-
Age (Years)		948	1.09 (1.06–1.12)	**< .001**
Gender	Female	468	0.99 (0.75–1.30)	0.92
	Male	480	-	-
Race/ethnicity	Hispanic	102	2.06 (1.29–3.30)	**0.003**
	Non-Hispanic Black	326	1.38 (0.97–1.95)	0.07
	Other	120	1.26 (0.79–2.02)	0.33
	Non-Hispanic White	317	-	-
Insurance type	Other	18	1.49 (0.54–4.12)	0.44
	Public	627	1.45 (1.07–1.98)	**0.018**
	Private	303	-	-
Premature	Yes	53	0.66 (0.34–1.28)	0.22
	No	895	-	-
Low Birth Weight	Yes	45	0.48 (0.22–1.05)	0.07
	No	903	-	-

**Table 3:** Multivariable association between obesity and ACE.

Covariate	Level	Odds Ratio	OR P-value
		(95% CI)	
ACE category	High ACE exposure	1.01 (0.70–1.46)	0.97
	Low ACE exposure	1.03 (0.68–1.55)	0.89
	No ACE exposure	-	-
Age (Years)		1.08 (1.05–1.12)	**< .001**
Race/ethnicity	Hispanic	2.04 (1.26–3.31)	**0.0038**
	Non-Hispanic Black	1.26 (0.88–1.82)	0.21
	Other	1.49 (0.92–2.41)	0.11
	Non-Hispanic White	-	-
Insurance type	Other	1.90 (0.64–5.64)	0.25
	Public	1.35 (0.95–1.91)	0.09
	Private	-	-
Low Birth Weight	Yes	0.44 (0.18–1.08)	0.07
	No	-	-

## Discussion

In this cross-sectional study consisting of 948 patients ages 2–19 years of age, the prevalence of high ACE exposure among overweight/obese participants (33%) was higher than that of non-obese participants (26%). Increased odds of obesity with high ACE exposure was also found among pediatric participants. However, this association attenuated and was no longer statistically significant after controlling for potential confounding variables (age, gender, race/ethnicity, insurance status, and birth weight).

The overall prevalence of ACE observed in this study is similar to other previous studies [22, 23]. However, it was higher than that reported by Caballero and colleagues and the difference could be due to the higher proportion of children immigrant families in their study, who have lower ACE than children in US native families [21].

Evidence for the negative impact of ACEs on weight status in children is inconsistent in the literature with most studies supporting an increased risk of obesity with ACE, albeit different experiences exert an influence at various timeframes during childhood and adolescence. In the Fragile Families and Child Wellbeing Study, a longitudinal study consisting of a cohort of 1,538 children born between 1998 and 2000 in urban hospitals, Schmeer found that children whose mothers dissolved a union or those with stable single mothers had an 80% higher risk of becoming overweight or obese between ages 3 and 5 years, as compared with children of stable married mothers [24]. Lynch *et al*. examined data from the 2011–2012 National Survey of Children’s Health and found that exposure to two or more adverse family experiences (AFEs) was associated with higher odds of overweight and obese status [25]. Heerman *et al* reported a similar result utilizing the 2011–2012 National Survey of Children’s Health. They found that children who had more AFEs were also at higher risk for overweight or obesity status [26]. Furthermore, Lynch *et al* found that exposure to certain childhood experiences, death of a parent and hardship due to family income, were stronger predictors of childhood obesity than other adverse experiences [25]. Isohookana *et al* observed that female adolescents who had experienced adverse childhood events were more likely to be obese and engage in unhealthy weight control behaviors [27]. However, all these previous studies did not control for birth weight, which is an important potential confounder [28, 29]. Boynton-Jarrett *et al* found that childhood exposure to maternal chronic intimate partner violence (IPV) correlated with an increased risk of obesity at age 5 years after adjusting for potential confounders including birth weight and socioeconomic status [23]. However, this association disappeared with early childhood exposure (less than 12 months of age) or late childhood exposure (at age 3 and/or 5 years) to maternal IPV suggesting that the timing of exposure might also be an important confounder. Future studies should consider these potential confounders and the timing of exposure.

The association between ACE and childhood health outcomes, including obesity, is biologically plausible and emerging evidence suggests that toxic stress, the accumulation or chronic activation of the body’s physiological stress response, is a potential mechanism [30]. Toxic stress exposure may manifest in the release of inflammatory cytokines, increased cortisol levels, and epigenetic modifications [25]. Kaufman *et al*. observed that ten methylation sites interact with ACEs to predict cross-sectional measures of BMI and an additional six sites were found to exert a main effect in predicting BMI in youth [25]. In addition to influencing biological pathways, early life stressors may affect child behavioral patterns, such as eating, sedentary behavior, and sleep, which could potentially result in increased childhood obesity [4, 5, 7–12, 31].

Frohlich *et al* found that a history of family adversity, maternal depression, and maternal attachment styles were important factors in predicting long-term success for a cohort of overweight and obese children ages 7 to 15 enrolled in a weight reduction program [32]. Lynch *et al* found that the probability of developing childhood obesity decreased when positive contextual factors were present, such as supportive neighborhoods and school safety [33]. Therefore, improving intervention programs and public policies focused on optimizing early environments may modify obesity risk in childhood.

Although several previous studies report an increased risk of obesity with ACEs, many of them relied on variable proxy measures, such as free/reduced lunch status, parental education level or place of residence to assess socioeconomic status, which could be a potential confounder or effect modifier [34, 35]. Min *et al* found that children living in poverty were more likely to engage in sedentary behaviors and have a poor BMI growth trajectory than children of higher socioeconomic status [36]. Other recent studies have also reported a higher ACEs prevalence among children of lower socioeconomic status [37–39]. In addition, previous studies show that birth weight is an important predictor of childhood obesity and therefore could be a potential confounder [28, 29]. Taken together, socioeconomic status and birth weight may be potential confounders or effect modifiers for the association between ACEs and childhood obesity and needs to be controlled in future studies. In addition, the timing of exposure could also play an important role in the association and warrants further investigation.

The present study has several limitations. It is a cross-sectional design and thus limits determination of causality. Additionally, our ACE measure does not ascertain important items, such as incarceration of a parent. Our study population included children and adolescence from a single institution and the findings may not be generalizable. Furthermore, it is difficult to discern the impact of ACE exposure over time on development of obesity in childhood. While multiple factors influencing development of obesity may be controlled for in a cross-sectional study design, longitudinal studies are better equipped to capture the effect of duration of ACE exposure on weight status during childhood [33]. Therefore, further prospective studies are warranted to explore the relationship between ACEs and obesity development in youth.

## Conclusion

The findings of this cross-sectional analysis may suggest an association between ACE and childhood obesity. However, the association attenuated after adjusting for age, race/ethnicity, insurance type, and birth weight. Larger prospective studies are warranted to better understand the association.

If an association actually exists, it could guide future interventions that may have a positive, life-long influence on a child’s overall health and well-being.
